# Patient characteristics and predictors of mortality in 470 adults admitted to a district general hospital in England with Covid-19

**DOI:** 10.1017/S0950268820002873

**Published:** 2020-11-24

**Authors:** J. V. Thompson, N. J. Meghani, B. M. Powell, I. Newell, R. Craven, G. Skilton, L. J. Bagg, I. Yaqoob, M. J. Dixon, E. J. Evans, B. Kambele, A. Rehman, G. Ng Man Kwong

**Affiliations:** Royal Oldham Hospital, Pennine Acute Hospitals NHS Trust, Rochdale Road, Oldham, Greater Manchester, OL1 2JH, UK

**Keywords:** Covid-19, mortality, outcomes, risk factors, SARS-CoV-2

## Abstract

Understanding risk factors for death from Covid-19 is key to providing good quality clinical care. We assessed the presenting characteristics of the ‘first wave’ of patients with Covid-19 at Royal Oldham Hospital, UK and undertook logistic regression modelling to investigate factors associated with death. Of 470 patients admitted, 169 (36%) died. The median age was 71 years (interquartile range 57–82), and 255 (54.3%) were men. The most common comorbidities were hypertension (*n* = 218, 46.4%), diabetes (*n* = 143, 30.4%) and chronic neurological disease (*n* = 123, 26.1%). The most frequent complications were acute kidney injury (AKI) (*n* = 157, 33.4%) and myocardial injury (*n* = 21, 4.5%). Forty-three (9.1%) patients required intubation and ventilation, and 39 (8.3%) received non-invasive ventilation. Independent risk factors for death were increasing age (odds ratio (OR) per 10 year increase above 40 years 1.87, 95% confidence interval (CI) 1.57–2.27), hypertension (OR 1.72, 95% CI 1.10–2.70), cancer (OR 2.20, 95% CI 1.27–3.81), platelets <150 × 10^3^/μl (OR 1.93, 95% CI 1.13–3.30), C-reactive protein ≥100 μg/ml (OR 1.68, 95% CI 1.05–2.68), >50% chest radiograph infiltrates (OR 2.09, 95% CI 1.16–3.77) and AKI (OR 2.60, 95% CI 1.64–4.13). There was no independent association between death and gender, ethnicity, deprivation level, fever, SpO_2_/FiO_2_, lymphopoenia or other comorbidities. These findings will inform clinical and shared decision making, including use of respiratory support and therapeutic agents.

## Introduction

Coronavirus disease 2019 (Covid-19) has spread rapidly around the world from an initial cluster of viral pneumonia linked to a wet market in Wuhan, China in December 2019. The United Kingdom (UK) recorded its first case on 31st January 2020 and as of 9th November 2020 has recorded 49 134 deaths representing the 12th highest per capita fatality rate worldwide at 73.9 per 100 000 people [1]. Oldham, a large town forming part of the Greater Manchester urban conurbation, has recorded the second highest incidence rate nationally, with 5005.7 cases per 100 000 people [2].

Much of the data outlining patient characteristics and risk factors for severe disease and mortality have come from Chinese case series with unadjusted analyses, and it is unclear how these findings relate to other populations. Increasing age or SOFA score and D-dimer >1 μg/ml were identified through multivariable analysis of 171 patients in Wuhan as independent predictors of death in hospital [3]. Other factors reported from China associated with severe disease or death includes hypertension, cardiovascular disease, chronic lung disease [4] and chronic kidney disease [5]. These factors were also identified by Docherty *et al*. as independent risk factors for death in an interim observational registry study of 20 133 hospitalised UK patients in addition to other chronic conditions [6]. Concern has been raised in the UK around the risk of death from Covid-19 in those of Black and Asian ethnicity, with these groups more likely to test positive and be hospitalised in the UK [7].

Understanding factors that predispose to death from Covid-19 in hospital is crucial to providing good patient care; including clinical and shared decision making, timely escalation of care for respiratory support and targeted use of potential antiviral or immunomodulatory therapies. The identification of modifiable risk factors would potentially allow mitigation of risk prior to infection. As such, we sought to describe the characteristics and outcomes of those admitted with Covid-19 in Oldham, Greater Manchester, UK, including sociodemographic factors, presenting clinical, biochemical and radiological features and complications. We assessed whether these characteristics were associated with death in hospital or within 30 days of discharge.

## Methods

### Study setting

The Royal Oldham Hospital is a 445 bed district general hospital located in Greater Manchester, a large urban conurbation in North West England serving a patient population of 230 000 people [8]. Oldham is amongst the most deprived areas in England, ranking 19th of 317 local authorities nationally in the 2009 indices of multiple deprivation (IMD) [9]. The predominant ethnic group is White (77.5%), with a considerably younger population of Pakistani (10.1%) and Bangladeshi (7.3%) heritage [10]. Population density is almost four times the national average at 15.8 persons per hectare [11]. Data were collected from electronic patient records in Symphony (EMIS Health) and HealthViews (Computer Sciences Corporation).

### Inclusion criteria

Four hundred and seventy patients were included. Individuals ≥18 years admitted to Royal Oldham Hospital were included if SARS-CoV-2 was detected by real-time polymerase chain reaction from a nasopharyngeal swab prior to or during admission. Patients included were admitted between 12th March 2020 and 19th May 2020, in addition to those who acquired Covid-19 as inpatients. Two patients were excluded due to missing data, with full datasets available for the remaining patients.

### Outcome measures

The main outcome measure of this retrospective observational cohort study was death during admission or within 30 days of discharge, as recorded in the electronic patient record. Deaths following discharge were notified automatically to the hospital electronic patient record.

### Explanatory variables

Age, gender, ethnicity, postcode, date of admission and date of discharge were recorded from structured fields. Age was categorised as <40 years, 40–50 years, 50–60 years, 60–70 years, 70–80 years or >90 years. Ethnicity was categorised into five groups – White British, Asian (including Indian, Pakistani, Bangladeshi, Chinese and other Asian background), African/Caribbean or other black background, Other white background and other/not specified. Postcodes were used to generate IMD quintiles and deciles using the UK government Ministry of Housing, Communities and Local Government Postcode Lookup tool.

Physiological parameters at admission including heart rate, systolic blood pressure, respiratory rate, oxygen saturation and temperature were collected from observations taken at triage in the emergency department. Fraction of inspired oxygen (FiO_2_) was estimated from oxygen flow rate using a standardised conversion table. The SpO_2_ (oxygen saturation)/FiO_2_ ratio, a validated surrogate for PaO_2_/FiO_2_, was categorised as >315, <315 or <235 as indicators of mild, moderate and severe acute lung injury (ALI), respectively [12]. Non-respiratory symptoms were categorised as gastrointestinal, cardiac, neurological and/or other incidental symptoms. Clinical frailty scale (CFS) [13] was recorded from admission documentation. Immunosuppression was defined as recent chemotherapy or use of immunomodulatory medication including oral steroids on admission.

The following laboratory parameters were collected: admission lymphocyte count ( × 10^3^/μl), lymphocyte nadir during admission ( × 10^3^/μl), admission platelet count ( × 10^3^/μl) and admission C-reactive protein (CRP) (μg/ml). Admission chest radiographs were categorised by degree of total new lung field infiltrates as none, less than 50%, or greater than 50%. Two clinicians reviewed radiographs where there was uncertainty.

Complications relating to Covid-19 were recorded, with pulmonary embolus, deep vein thrombosis and stroke recorded if confirmed by imaging studies. Super-added bacterial pneumonia was recorded if a causative organism was isolated. Acute kidney injury (AKI) was assessed using the Kidney Disease Improving Global Outcomes (KDIGO) criteria, and myocardial injury was deemed present if troponin elevation above the normal range (<2 ng/l) was present without alternative explanation.

### Statistical analysis

Data were collected using Microsoft Excel. STATA 13 was used to produce summary statistics and assess associations with death in hospital or within 30 days of discharge.

For univariate analysis Pearson's chi-squared test (*χ*^2^) was used to test the significance of categorical variables. Odds ratios (ORs) for death and 95% confidence intervals (CIs) were calculated for categorical variables. A K-sample equality-of-medians test was used to assess for significant differences in the medians of continuous variables for survivors compared to non-survivors for non-normally distributed variables, and a two-sided *t* test was used to assess for significant differences in means of normally distributed variables. A significance level of 0.05 was used for all statistical tests. ORs for continuous variables are per 1 unit increase unless otherwise specified.

A logistic regression model to examine which factors predict death was constructed using dependent variables previously proposed as poor prognostic markers, as well as those with *p* values from univariate statistical tests of <0.25. The initial model was refined through backward elimination using the likelihood ratio test to assess for significant difference in model fit. CFS was removed from the model due to collinearity with age. The linktest program within STATA did not provide evidence for a model specification error. Goodness of fit for the final model deemed satisfactory using McFadden's pseudo-*R*^2^ test.

### Ethical approval

This study was approved by the Northern Care Alliance (NCA) Research Management and Support (RM&S) team and did not require the approval from the NHS Health Research Authority (HRA).

## Results

### Sociodemographic factors

**Fig. 1.**
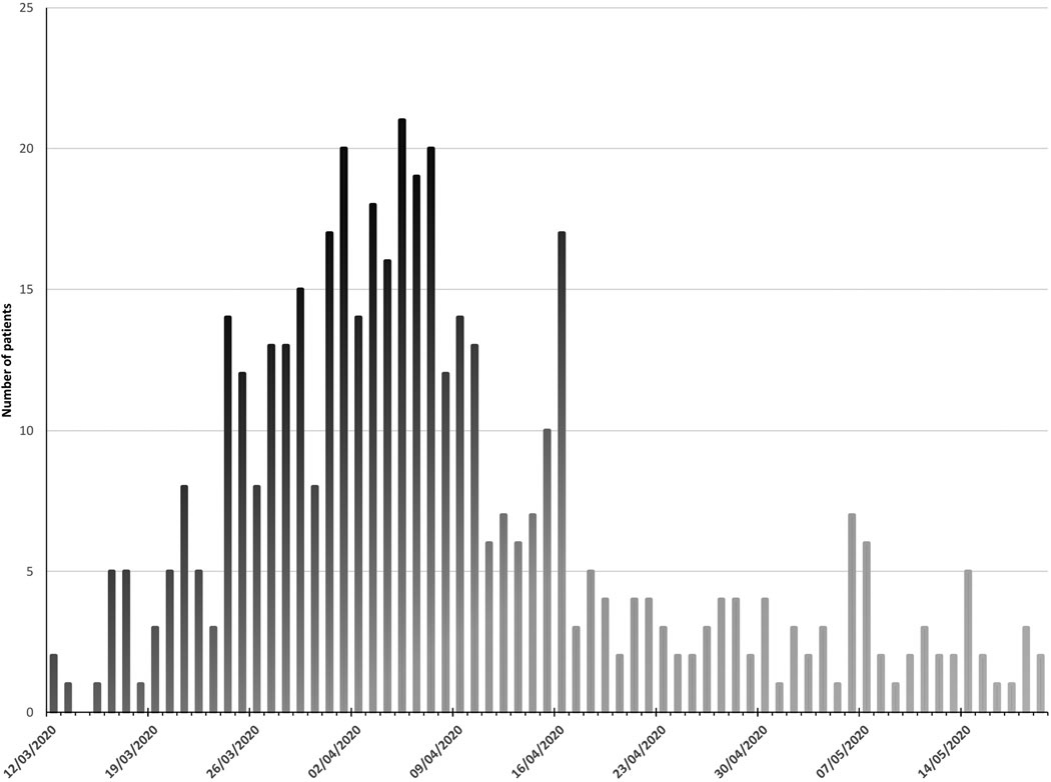
Patients admitted with Covid-19 to Royal Oldham Hospital, UK by date.

**Fig. 2.**
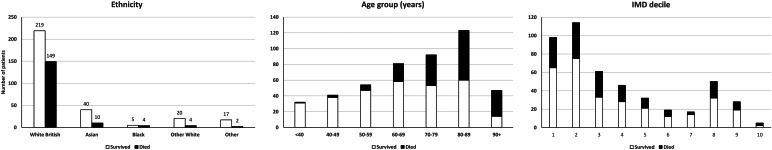
Sociodemographic characteristics of patients admitted with Covid-19 to Royal Oldham Hospital, UK stratified by survival to 30 days post-discharge. IMD, index of multiple deprivation, where the first decile represents highest level of deprivation.

Of 470 patients admitted 36.0% (*n* = 169) died during admission or within 30 days of discharge (Figure 1). The median age of those admitted was 71 years (interquartile range (IQR) 57–82 years), and the median age of non-survivors was 80 years. More men (54.3%, *n* = 255) than women (45.7%, *n* = 215) were admitted but following admission no difference in frequency of death was present (*P* = 0.95). White British patients were the largest ethnic group (78.3%, *n* = 368), followed by Asian (10.6%, *n* = 50) and Other White background (5.1%, *n* = 24), predominantly from Eastern Europe. In keeping with high local levels of deprivation, 44.9% of patients were classified as being in the most deprived quintile nationally. Death was significantly more frequent with increasing age (per 10 year age bracket increase above age 40 years OR 1.89, 95% CI 1.62–2.20, *P* < 0.001), and less frequent among those of Asian (OR 0.37, 95% CI 0.18–0.76, *P* < 0.01) and Other white background ethnicity (OR 0.29, 95% CI 0.10–0.88, *P* < 0.02) compared to White British patients. IMD quintile was not associated with death (Table 1; Fig. 2).

**Table 1.** Sociodemographic characteristics of patients admitted with Covid-19 stratified by survival

Sociodemographic characteristic	Total number of subjects (% of all patients)	Survival to 30 days post discharge	Death during admission or within 30 days of discharge	OR for death (95% CI)	*P* value
All patients	470 (100%)	301 (64.0%)	169 (36.0%)		
Mean age (s.d.)	68.7 (17.4)	63.4 (17.6)	78.3 (12.1)	1.07^a^ (1.05–1.08)	<0.001
Median age (IQR)	72 (57–82)	65 (51–78)	80 (72–87)		
Age group					
<40 years	32 (6.8%)	31 (10.3%)	1 (0.6%)	1.00	<0.001
40–49 years	41 (8.7%)	38 (12.6%)	3 (1.8%)	2.44 (0.24–24.71)	
50–59 years	54 (11.5%)	47 (15.6%)	7 (4.1%)	4.62 (0.54–39.39)	
60–69 years	81 (17.2%)	58 (19.3%)	23 (13.6%)	12.29 (1.58–95.40)	
70–79 years	92 (19.6%)	53 (17.6%)	39 (23.1%)	22.81 (2.98–174.35)	
80–89 years	123 (26.1%)	60 (19.9%)	63 (37.3%)	32.55 (4.31–245.98)	
≥90 years	47 (10.0%)	14 (4.6%)	33 (19.5%)	73.07 (9.06–589.09)	
Gender					
Female	215 (45.7%)	138 (45.9%)	77 (45.6%)	1.00	
Male	255 (54.3%)	163 (54.1%)	92 (54.4%)	1.01 (0.69–1.48)	0.95
Ethnicity					
White British	368 (78.3%)	219 (72.8%)	149 (88.1%)	1.00	0.001
Asian	50 (10.6%)	40 (13.3%)	10 (5.9%)	0.37 (0.18–0.76)	
Black African, Caribbean or other black background	9 (1.9%)	5 (1.7%)	4 (2.4%)	1.18 (0.31–4.45)	
Other White Background	24 (5.1%)	20 (6.6%)	4 (2.4%)	0.29 (0.10–0.88)	
Other/Not specified	19 (4.0%)	17 (5.6%)	2 (1.2%)	0.17 (0.04–0.76)	
IMD quintile					
5 (least deprived)	33 (7.0%)	21 (7.0%)	12 (7.1%)	1.00	0.56
4	67 (14.2%)	46 (15.3%)	21 (12.4%)	0.80 (0.33–1.92)	
3	51 (10.9%)	33 (11.0%)	18 (10.7%)	0.95 (0.38–2.38)	
2	108 (23.0%)	62 (20.6%)	46 (27.2%)	1.30 (0.58–2.90)	
1 (most deprived)	211 (44.9%)	139 (46.2%)	72 (42.6%)	0.91 (0.42–1.95)	

a per 1 year increase.

### Comorbidities

**Fig. 3.**
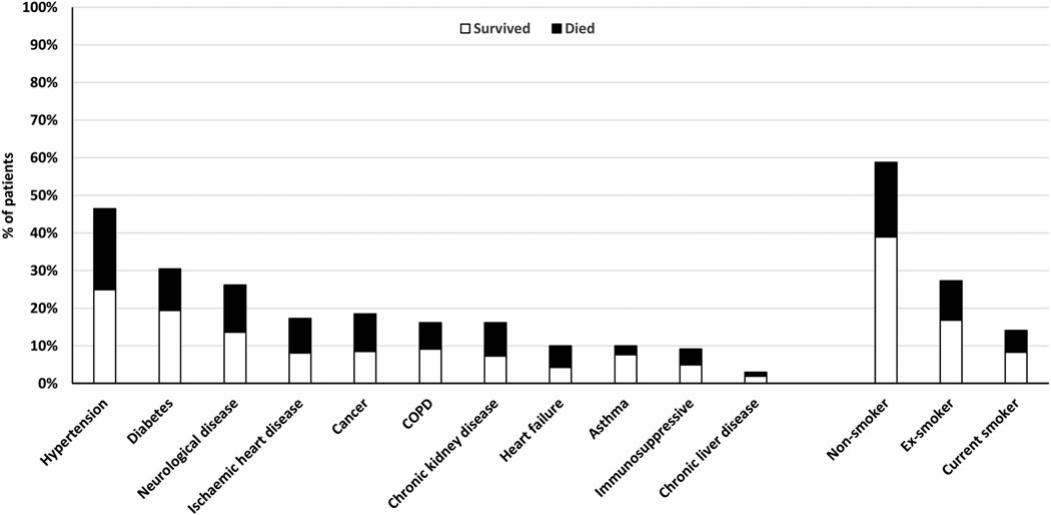
Comorbidities of patients admitted with Covid-19 to Royal Oldham Hospital, UK stratified by survival to 30 days post-discharge.

Median CFS was significantly higher in non-survivors (six IQR 4–7 *vs.* three IQR 2–5 for survivors), with a mean total number of significant comorbidities of 3.2 (s.d. 1.7) among those who died *vs.* 2.1 (s.d. 1.6) for survivors. In total, 13.9% (*n* = 66) had no prior significant medical conditions. The most common comorbidities were hypertension (46.4%, *n* = 218), diabetes (30.4%, *n* = 143) and chronic neurological disease including dementia and other neurodegenerative conditions (26.1%, *n* = 123). Hypertension, ischaemic heart disease, heart failure, chronic kidney disease, chronic neurological disease and current or prior cancer were significantly more prevalent among those who died. Smoking, diabetes, chronic obstructive pulmonary disease (COPD), asthma, chronic liver disease and immunosuppressive medication on admission were not significantly more common among non-survivors (Table 2; Figs 1 and 3).

**Table 2.** Comorbidities of patients admitted with Covid-19 stratified by survival

Comorbidity	Total number of subjects (% of all patients)	Survival to 30 days post discharge	Death during admission or within 30 days of discharge	OR for death (95% CI)	*P* value
Median CFS	4 (IQR 3–6)	3 (IQR 2–5)	6 (IQR 4–7)	1.72 (1.52–1.94)	<0.001
Mean total number of significant comorbidities	2.5 (s.d. 1.7)	2.1 (s.d. 1.6)	3.2 (s.d. 1.7)	1.45 (1.31–1.67)	<0.001
Smoking status					
Non-smoker	276 (58.7%)	183 (60.8%)	93 (55.0%)	1.00	0.45
Ex-smoker	128 (27.2%)	79 (26.2%)	49 (29.0%)	1.22 (0.79–1.89)	
Current smoker	66 (14.0%)	39 (13.0%)	27 (16.0%)	0.51 (0.79–2.36)	
Comorbidity					
Hypertension	218 (46.4%)	117 (38.9%)	101 (55.0%)	2.34 (1.49–3.43)	<0.001
Diabetes	143 (30.4%)	91 (30.2%)	52 (30.8%)	1.03 (0.68–1.54)	0.90
Ischaemic heart disease	81 (17.2%)	38 (12.6%)	43 (25.4%)	2.36 (1.45–3.84)	<0.01
Heart failure	47 (10%)	20 (6.6%)	27 (16.0%)	2.67 (1.45–4.93)	<0.01
COPD	76 (16.1%)	43 (14.3%)	33 (19.5%)	1.46 (0.88–2.40)	0.16
Asthma	47 (10.0%)	36 (12.0%)	11 (6.5%)	0.51 (0.25–1.04)	0.06
Chronic kidney disease	76 (16.1%)	34 (11.3%)	42 (24.9%)	2.60 (1.56–4.28)	<0.001
Chronic neurological disease	123 (26.1%)	64 (21.3%)	59 (34.9%)	1.99 (1.31–3.02)	<0.001
Chronic liver disease	14 (3.0%)	9 (3.0%)	5 (3.0%)	0.99 (0.33–3.00)	0.99
Current or previous cancer	87 (18.5%)	40 (13.3%)	47 (27.8%)	2.51 (1.57–4.04)	<0.001
Current use of immunosuppressive medication	43 (9.1%)	23 (7.6%)	20 (11.8%)	1.62 (0.86–3.05)	0.13

### Physiological parameters on admission

Tachypnoea was common, with 70% (*n* = 329) presenting with a respiration rate ≥20/min, and 38.5% (*n* = 171) required supplemental oxygen. In total, 15.7% (*n* = 74) of patients were classified by the SpO_2_/FiO_2_ index as severe ALI, 14.3% (*n* = 67) as moderate ALI and 70% (*n* = 329) as mild or no ALI. Fever (temperature ≥37.8 °C) was present in 40.6% (*n* = 191) at triage. Admission respiration rate, severity of ALI, heart rate, blood pressure or fever were not associated with death.

The median duration of symptoms prior to presentation was 4 days (IQR 2–7), with no significant difference between survivors and non-survivors. Gastrointestinal symptoms (abdominal pain, nausea, vomiting or diarrhoea) were present in 18.9% (*n* = 89) of patients, neurological symptoms (confusion or focal neurological signs) were present in 7.9% (*n* = 37) patients, and presentations in keeping with cardiac disease (acute coronary syndrome and myocarditis) were evident in 1.3% (*n* = 6) patients. Interestingly, one patient presented with posterior reversible encephalopathy syndrome, and one with cerebral vasculitis. In total, 10.4% of patients (*n* = 49) presented with clinical features consistent with alternative diagnoses in whom Covid-19 was found incidentally or diagnosed during admission (Table 3).

**Table 3.** Clinical characteristics of patients admitted with Covid-19 stratified by survival

Clinical characteristic	Total number of subjects (% of all patients)	Survival to 30 days post discharge	Death during admission or within 30 days of discharge	OR for death (95% CI)	*P* value
Physiological parameters at triage					
Median SpO_2_/FiO_2_ index	442 (IQR 278 −462)	428 (IQR 475–457)	443 (IQR 278–462)	0.999 (0.997–1.001)	0.24
Median respiration rate	21 (IQR 19–26)	20 (IQR 18–25)	22(IQR 19–26)	1.01 (0.99–1.04)	0.35
Median heart rate	90 (IQR 78–106)	92 (IQR 79–107)	90 (IQR 77–106)	1.00 (0.99–1.01)	0.54
Median blood pressure	129 (IQR 115–146)	131 (IQR 114–148)	128 (IQR 116–146)	1.00 (0.99–1.00)	0.78
No fever	279 (59.3%)	177 (58.8%)	102 (60.3%)		
Fever (≥37.8 °C)	191 (40.6%)	124 (41.2%)	67 (39.6%)	0.94 (0.64–1.38)	0.74
Median duration of symptoms at presentation (days)	4 (IQR 2–7)	5 (IQR 2–7)	3 (IQR 1–7)	0.99 (0.95–1.02)	0.43
GI symptoms (nausea, vomiting, diarrhoea and abdominal pain)	89 (18.9%)	54 (17.9%)	35 (20.7%)	1.19 (0.74–1.92)	0.46
Neurological symptoms (confusion or focal neurological signs)	37 (7.9%)	25 (8.3%)	12 (7.1%)	0.84 (0.41–1.73)	0.64
Cardiac presentation (ACS/myocarditis)	6 (1.3%)	4 (1.3%)	2 (1.2%)	0.89 (0.16–4.90)	0.89
Presentation consistent with possible Covid-19	421 (89.6%)	265 (88.0%)	156 (92.3%)		
Presentation not consistent with Covid-19^a^	49 (10.4%)	36 (12.0%)	13 (7.7%)	0.61 (0.32–1.19)	0.32

a SARS-2 CoV identified incidentally at presentation or during admission.

### Biochemical and radiological features on admission

In total, 54.9% (*n* = 258) of patients had a falling lymphocyte count during admission, with a median value on admission of 1.0 (IQR 0.7–1.4) and a median lymphocyte nadir of 0.7 (IQR 0.5–1.1). There was no significant difference in these values between survivors and non-survivors, and falling lymphocyte count during admission was not associated with death. The median admission CRP was significantly higher among those who died (94 μg/ml IQR 38–167 μg/ml *vs.* 75 μg/ml IQR 34–132 μg/ml for survivors), while the median admission platelet count was significantly lower among non-survivors (190 × 10^3^/μl, IQR 142–275 × 10^3^/μl *vs.* 224 × 10^3^/μl, IQR 178–302 × 10^3^/μl for survivors). New infiltrates were noted on admission chest radiographs of 63.4% (*n* = 298) of patients, with more than 50% total lung field involvement evident in 25.5% (*n* = 120). Univariate analysis did not reveal an increased risk of death among those with new chest radiograph infiltrates (Table 4).

**Table 4.** Biochemical and radiological characteristics of patients admitted with Covid-19 stratified by survival

Biochemical and radiological features	Total number of subjects (% of all patients)	Survival to 30 days post discharge	Death during admission or within 30 days of discharge	OR for death (95% CI)	*P* value
Median admission lymphocyte count ( × 10^3^/μl)	1.0 (IQR 0.7–1.4)	1.0 (IQR 0.7–1.5)	0.9 (IQR 0.6–1.3)	1.01 (0.99–1.04)	0.20
Median lymphocyte nadir ( × 10^3^/μl)	0.7 (IQR 0.5–1.1)	0.8 (IQR 0.5–1.2)	0.7 (IQR 0.4–1.0)	1.01 (0.98–1.04)	0.46
Fall in lymphocyte count during admission	258 (54.9%)	159 (52.8)	99 (58.6)	1.26 (0.86–1.85)	0.23
Median admission CRP (μg/ml)	80.3 (IQR 34–143)	75 (IQR 34–132)	94 (IQR 38–167)	1.002 (1.000–1.005)	0.05
Admission CRP ≥100 μg/ml	194 (49.3%)	113 (37.5%)	81 (47.9%)	1.53 (1.05–2.24)	0.03
Median admission platelet count ( × 10^3^/μl)	218 (IQR 162–297)	224 (IQR 178–302)	190 (IQR 142–275)	0.99 (0.99–1.00)	0.05
Admission platelet count <150 × 10^3^/μl	98 (20.9%)	49 (16.3%)	49 (28.9%)	2.10 (1.34–3.30)	<0.001
Chest radiograph findings on admission					
Normal	172 (36.6%)	116 (38.5%)	56 (33.1%)	1.00	0.20
<50% total lung field infiltrates	178 (37.8%)	116 (38.5%)	62 (36.7%)	1.10 (0.71–1.73)	
>50% total lung field infiltrates	120 (25.5%)	69 (22.9%)	51 (30.2%)	1.53 (0.95–2.48)	

### Complications and respiratory support

The most commonly observed complications due to Covid-19 were AKI (33.4%, *n* = 157), myocardial injury (4.5%, *n* = 21) and bacterial pneumonia (3.2%, *n* = 15). Pulmonary embolus was identified on 5 of 24 computed tomography pulmonary angiograms (1.1% of all patients), while deep vein thrombosis was identified from 8 of 19 duplex venous ultrasounds (1.7% of all patients). AKI and myocardial injury were more common among those who died.

Respiratory support was required by 14.7% (*n* = 69) of patients, with 1.7% (*n* = 8) of patients receiving high flow nasal cannula (HFNC), 8.3% (*n* = 39) receiving non-invasive continuous positive pressure airway ventilation (CPAP) and 9.2% (*n* = 43) intubated and mechanically ventilated (IMV). The use of more than one mode of respiratory support was frequently employed (Table 5).

**Table 5.** Complications and respiratory support for patients admitted with Covid-19 stratified by survival

Respiratory support and complications	Total number of subjects (% of all patients)	Survival to 30 days post discharge	Death during admission or within 30 days of discharge	OR for death (95% CI)	*P* value
Mode of respiratory support					
CPAP	39 (8.3%)	26 (8.6%)	13 (7.7%)	0.88 (0.44–1.76)	0.72
HFNC	8 (1.7%)	3 (1.0%)	5 (3.0%)	3.02 (0.71–12.8)	0.13
IMV	43 (9.1%)	28 (9.3%)	15 (8.8%)	0.95 (0.49–1.83)	0.88
Complications during admission					
Pulmonary embolism	5 (1.1%^a^)	4 (1.3%)	1 (0.6%)	0.44 (0.05–3.99)	0.47
Deep vein thrombosis	8 (1.7%^b^)	5 (1.7%)	3 (1.8%)	1.07 (0.25–4.53)	0.93
Myocardial Injury	21 (4.5%)	9 (3.0%)	12 (7.1%)	2.48 (1.02–6.01)	0.04
AKI	157 (33.4%)	72 (23.9%)	85 (50.3%)	3.20 (2.15–4.81)	<0.001
Stroke	1 (0.2%)	1 (0.3%)	0 (0%)	N/A	
Bacterial pneumonia	15 (3.2%)	12 (4.0%)	3 (1.8%)	0.43 (0.12–1.56)	0.20

a 1.1% of all patients, 20.8% of those who underwent CTPA.

b 1.7% of all patients, 42.1% of those who under venous duplex ultrasound.

### Multivariate analysis

**Fig. 4.**
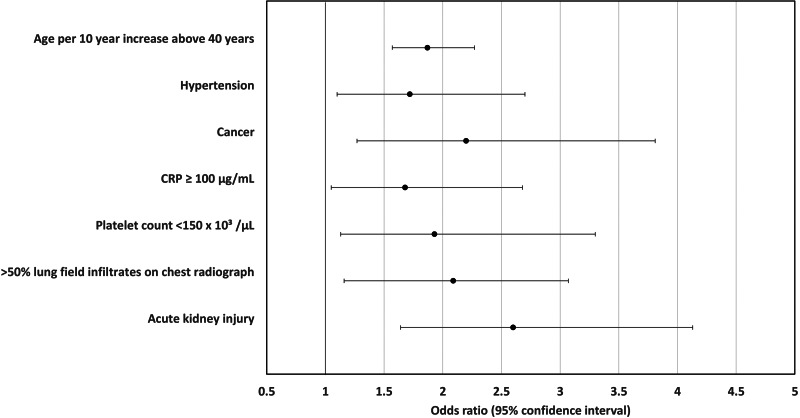
Independent predictors of death for patients admitted with Covid-19 to Royal Oldham Hospital, UK. All risk factors assessed on admission to hospital with the exception of AKI which was classified as a complication of Covid-19 and recorded if present at any time during admission.

Logistic regression identified the following variables which were also significant in univariate analysis as independent risk factors for death – increasing age, history of hypertension, current or previous cancer, admission CRP ≥100 μg/ml, admission platelet count <150 × 10^3^/μl and AKI. Additionally, the model identified >50% total lung field infiltrates, which was not significant in univariate analysis, as a significant risk factor for death. Ethnicity, history of chronic kidney disease, ischaemic heart disease, heart failure or chronic neurological disease and myocardial injury, which were observed more frequently among those who died, were not significant in the adjusted model. No other explanatory variables were found to be significant independent predictors of death (Table 6; Fig. 4).

**Table 6.** Significant risk factors for death among patients admitted with Covid-19 – univariate and multivariate logistic regression

Risk factor	Univariate OR for death compared to reference group	95% CI	*P* value (Wald test)	Adjusted OR for death compared to reference group (logistic regression)	95% CI	*P* value (Wald test)
Age – per 1 year increase	1.07	1.05–1.08	<0.001			
Age – per 10 year increase above age 40	1.89	1.62–2.20	<0.001	1.87	1.57–2.27	<0.001
Ethnicity						
White British	1.00					
Asian	0.37	0.18–0.76	<0.01			
Black African, Caribbean or other black background	1.18	0.31–4.45	0.81			
Other White Background	0.29	0.10–0.88	0.03			
Other/Not specified	0.17	0.04–0.76	0.02			
Comorbidities						
Median CFS^a^	1.72	1.52–1.94	<0.001	Not included in regression model due to collinearity with age		
Total number of significant comorbidities^a^	1.45	1.31–1.67	<0.001			
Hypertension	2.34	1.49–3.43	<0.001	1.72	1.10–2.70	0.02
Chronic kidney disease	2.60	1.56–4.28	<0.001			
Ischaemic heart disease	2.36	1.45–3.84	<0.01			
Heart failure	2.67	1.45–4.93	<0.01			
Chronic neurological disease	1.99	1.31–3.02	<0.001			
Cancer – current or previous	2.51	1.57–4.04	<0.001	2.20	1.27–3.81	<0.01
Biochemical						
Admission CRP ≥100 μg/ml	1.53	1.05–2.24	0.03	1.68	1.05–2.68	0.03
Admission platelet count <150 × 10^3^/μl	2.10	1.34–3.30	<0.001	1.93	1.13–3.30	0.01
Admission chest radiograph						
Normal	1.00					
<50% total lung field infiltrates	1.10	0.71–1.73	0.65			
>50% total lung field infiltrates	1.53	0.95–2.48	0.08	2.09	1.16–3.77	0.01
Complications						
Myocardial injury	2.48	1.02–6.01	0.04			
AKI	3.20	2.15–4.81	<0.001	2.60	1.64–4.13	<0.001

a Per 1 unit increase.

## Discussion

### Summary of main findings and comparison to other studies

We assessed a wider range of potential risk factors for death than previous studies, with selection of variables guided by their clinical importance to us as hospital physicians in describing the overall clinical state of patients admitted to hospital with acute illness. In keeping with prior studies which adjusted for either no or fewer clinically relevant factors [3, 4, 6], we identified a wide range of factors that were more common among non-survivors. However, only increasing age, hypertension, previous or current cancer, platelet count <150 × 10^3^/μl, CRP ≥100 μg/ml, greater than 50% total lung field infiltrates on chest radiograph and AKI were identified as independent risk factors for death in our clinically relevant and statistically robust regression model. Our mortality rate of 36.0% (*n* = 169) is in keeping with the UK's mortality rate for those hospitalised with Covid-19 [14].

More men than women were admitted, although the 53.2% of men in our cohort is significantly smaller than the 60% of males admitted in a previous large UK multicentre study [6]. Incredibly, we observed no difference in mortality by gender, despite men accounting for 80% of those requiring intubation and gender remained a non-significant factor following multivariate analysis. This finding is in stark contrast to reports that men hospitalised with Covid-19 are at higher risk of death [6]. Although death was more frequent among White British patients, no ethnic group was an independent risk factor for mortality. Ethnicity was strongly attenuated in the logistic regression model by age, with the median age of White British patients 20 and 31 years greater than those of Black and Asian patients respectively. This study was underpowered to detect a mortality difference in Black patients given the low number admitted.

Hypertension, diabetes, cancer and current smoking were recorded far more frequently in our cohort than in the largest UK study assessing the characteristics of those admitted with Covid-19 [6]. Hypertension was present in almost half of those admitted and 46.3% (*n* = 101) of this group died suggesting patients with hypertension should be regarded as a particularly high-risk group. Although hypertension and cancer were independent risk factors for death in our multivariate analysis [6], previously reported factors including diabetes, COPD and ischaemic heart disease [3, 15] were not found to be independent risk factors. The majority of patients admitted had at least one comorbidity, and only four patients without comorbidity died, suggesting that those without significant medical problems are at very low risk of death.

Lymphopoenia was observed in 63.2% of patients, in keeping with previous studies suggesting it is an important predictor of severe disease [16]. However, we did not find any evidence that admission lymphocyte count, lymphocyte nadir or falling lymphocytes are associated with death in either univariate or multivariate analysis, suggesting that any prognostic implication is rendered prior to the point of hospital admission. We suggest that lymphocyte count is of diagnostic utility only and is not a useful prognostic marker during hospital admission. However, we identified CRP ≥100 μg/ml and platelet count <150 × 10^3^/μl as independent risk factors for death, which could be monitored serially to risk stratify patients.

SpO_2_/FiO_2_ is a validated surrogate for PaO_2_/FiO_2_ used to classify severity of acute respiratory distress syndrome [12], a condition thought to share pathophysiological mechanisms with Covid-19. Surprisingly, no difference in SpO_2_/FiO_2_ was observed between survivors and non-survivors, but the presence of infiltrates involving more than 50% of the lung fields on admission chest radiograph was an independent predictor of death. We recommend greater prognostic weight be given to severity of radiological changes than SpO_2_ and oxygen requirement at presentation.

Optimal fluid management is imperative given the high incidence of AKI and independent association with death in our cohort, underlining a recent recommendation to maintain euvolaemia from the UK National Institute for Health and Care Excellence (NICE) [17]. This followed initial concerns that fluid therapy may increase mortality through worsening of non-cardiogenic pulmonary oedema. It is unclear how changing practice has impacted upon the incidence of AKI and its attributable mortality.

### Strengths

We assessed variables available on admission that we as clinicians felt were important in describing the clinical state of acutely unwell patients. Our results are of particular relevance to areas of similar sociodemographic makeup including cities in the UK, and overseas. Unlike previous studies, we report outcomes for all included patients. We utilised an iterative process (backward elimination) to derive a fully adjusted model with good fit to our dataset.

### Limitations

We assessed risk factors for death present in hospitalised patients and have not investigated those not requiring admission. Consequently, there may be risk factors that confer risk for hospitalisation, and therefore a higher risk of death, that we have not identified. This is suggested, for example, by the disproportionate number of men admitted. We did not utilise admission criteria, with decision to admit at clinician discretion. As such, patients with risk factors for death identified in previous studies may have been admitted to the hospital more readily than those thought to be at lower risk of death. The impact of this upon the associations identified in this study is attenuated by the fact that the majority of patients who die from Covid-19 in the UK do so in hospital.

There is uncertainty over the best method for variable selection in logistic regression modelling. All independent variables initially collected in this study were deemed to be of importance in describing the overall state of acutely unwell patients with respiratory infection, and previously suggested risk factors for death from Covid-19 included where routinely collected. Variables that were highly unlikely to be of significance in the final model were removed prior to backward stepwise selection to minimise the risk of including redundant variables in the final model and reduce model complexity. Backward stepwise selection was chosen for the final stage of variable selection such that variables with small but statistically significant contributions to mortality risk were identified, which may have been overlooked by forward or stepwise selection processes. All variables included in the final model have strong theoretical rationale for inclusion. For example, pre-existing endothelial dysfunction secondary to hypertension may predispose patients to endotheliitis and subsequent increased risk of death from Covid-19 [18].

## Conclusions

We characterised the ‘first wave’ of patients presenting to a district general hospital with Covid-19 in one of England's highest incidence areas, and determined that increasing age, hypertension, cancer, CRP >100 μg/ml, platelets <150 × 10^3^/μl, severe chest radiograph appearance and AKI independently predict death following admission. We propose greater prognostic weight be given to these factors than to SpO_2_, oxygen requirement or lymphopoenia, which did not predict death. Results from both our unadjusted and adjusted analyses dispute other previously proposed risk factors for death in hospital including male gender and diabetes.

These findings have the potential to inform decision making by front-line clinicians at the point of admission to hospital allowing risk stratification, improved shared decision-making with patients, the timely provision of respiratory support and targeted use of potential therapies for those most likely to benefit. If our model is prospectively validated, we anticipate introducing a clinical decision tool to aid prognostication for hospitalised patients with Covid-19.

## Data Availability

Data are available directly by contacting the corresponding author, pending institutional approval.
